# Why do some pregnant women prefer cesarean delivery in first pregnancy?

**Published:** 2013-04

**Authors:** Ali Gholami, Shaker Salarilak

**Affiliations:** 1 *School of Nursing, Neyshabur University of Medical Sciences, Neyshabur, Iran.*; 2 *Department of Public Health, Faculty of Medicine, Islamic Azad University, Tabriz Branch, Tabriz, Iran.*

**Keywords:** *Cesarean delivery*, *Vaginal delivery*, *Pregnancy*, *Patient preference*

## Abstract

**Background: **The increasing number of cesarean section is a great concern in many countries. In Iran cesarean section rate has been steadily rising from 35% in 2000 to 40% in 2005. Preferences for cesarean are often associated with some factors.

**Objective:** To investigate factors associated with preference for cesarean delivery, with special emphasis on pregnant women’s preferences in first pregnancy in Neyshabur (Northeast of Iran).

**Materials and Methods: **In this cross-sectional study, written questionnaires were completed via face to face interview with 797 pregnant women in first pregnancy. Socio-demographic data, preference toward mode of delivery and factors associated with it were assessed by applying questionnaire. Univariate and multivariate analysis were performed to identify the independent variables associated with preference for cesarean delivery.

**Results:** In this study observed that 18.6% of pregnant women preferred caesarean delivery in first pregnancy. The mean age of pregnant women that they preferred cesarean delivery was upper than pregnant women that they preferred vaginal delivery and this difference was statistically significant (p=0.006). There was a statistically significant relation between preference for cesarean delivery and the following variables: educational level (p<0.001), gestational age (p=0.003) spouse’s age of pregnant women (p=0.001), physician’s advice (p<0.001), and fear of delivery (p<0.001).

**Conclusion:** The results of this study show that the majority of pregnant women do not prefer caesarean delivery to vaginal delivery. Nevertheless the preference rate for cesarean delivery exceeded 15% that suggested by WHO and most important factors in pregnant women prefer cesarean deliveries are fear of delivery and physician’s advice.

## Introduction

Cesarean Section (CS) rates around the world have been increasing (1-8). International concerns over such increases have prompted the World Health Organization (WHO) to suggest that CS rates should not exceed 15% (9). Cesarean delivery (CD) rates have been a major concern of health policy makers in many developed and developing countries (10). 

CD as an alternative procedure for child delivery is an invasive and risk-bearing medical practice involving abdominal surgery and has considerable drawbacks, including postoperative pain, higher delivery cost, prolonged hospital stay, neonatal respiratory distress, and delay in breast feeding initiation, CD have some benefits, for example avoidance of an emergency delivery, prevention of some term demises, decreased transmission of HIV and other infections, and decrease in birth related injuries (11-16). 

Women’s requests for CS have, to a great extent, attributed to the escalating rate. CS on maternal request is planned surgery performed without medical indication, where the wish of the woman compensates for the lack of medical reasons. The concept of “patient’s choice” is well accepted among obstetricians (17, 18). The decision to perform a primary CS has important implications for maternal morbidity in the current pregnancy and mode of delivery and maternal morbidity in subsequent pregnancies (19-21). 

Many efforts have been made to identify the factors that contribute to CD. Researchers have documented the role of clinical factors (previous CD, dystocia, fetal distress, breech presentation, and mal presentation) and no clinical factors (socioeconomic status, race, maternal age, institutional characteristics, physician practice styles, and other characteristics) in CD (22, 23). 

Preferences for cesarean are often associated with some factors such as having a history of previous CD, fear of birth, maternal age, maternal education, socioeconomic factors and so on (24-32). Having a history of delivery may affect the preference for cesarean in pregnant women (especially in those who have a history of CD) but we want to study pregnant women without this factor, so the aim of this study was to investigate some factors associated with preference for CD in Northeast of Iran (Neyshabur), with special emphasis on pregnant women’s preferences in first pregnancy.

## Materials and methods

This investigation is a cross-sectional study that was conducted on the pregnant women without previous pregnancy in Neyshabur (Northeast of Iran). In this study of 1780 pregnant women in studied period (February 2011 to March 2011), 983 were excluded from the study, 76 because of disagreement to contribute in study and 907 because they had previous pregnancy. Accordingly, 797 pregnant women remained for analysis. All subjects gave informed consent to participate in the study.

**Procedure and study Instrument**

Data collection was formed via face-to-face interview with pregnant women who agreed to participate in this study and for enhance accuracy; all participants were informed that their responses would remain confidential. Questionnaire of this study was adapted and elaborated from questionnaires used in other studies that focused on preference toward mode of delivery and the etiology of these preferences in pregnant women (10, 11, 31, 33-41). This questionnaire contained two parts. 

The first part of the questionnaire aimed to collect information on the socio-demographic data of the respondents and the second part sought information on preference toward mode of delivery and factors associated with it. The questionnaire was pilot tested at a health center in Neyshabur, and revisions were made to ensure validity of it. From February 20, 2011 to March 20, 2011, the questionnaires were completed for total pregnant women in first pregnancy (797 persons) at all health centers in Neyshabur. 

Inclusion criteria to study included: (a) women in first pregnancy (b) women who were pregnant at any time from February2011 to March 2011, (c) residence in Neyshabur, (d) women’s agreement. Exclusion criteria included any circumstances against inclusion criteria. 

**Dependent and independent variables**

Preference toward mode of delivery was considered as dependent variable. The other data collected were age, educational level, occupation, fear of delivery, gestational age in pregnant women and age, educational level, occupation in spouse of pregnant women as well as local residence, safety of the baby, physician's advice and planned pregnancy as independent variables.

**Statistical analysis**

The data analysis was performed using the Statistical Package for the Social Sciences (SPSS) for Windows version 16.0 (SPSS Inc, Chicago, IL, USA). Descriptive analyses performed including frequencies, percentages, ranges, means, and standard deviations. In this study logistic regression model was used to investigate the relation between women’s preference toward mode of delivery and independent variables. We reported Odds Ratio (OR) with 95% confidence interval (CI). Various factors tested to have an association with preference for mode of delivery with p<0.05.

## Results

The characteristics of study population are shown in Table I. The mean age of the study participants was 23.96±4.45 years (Range: 14-44). Of all pregnant women who contributed in this study, 649 (81.4%) said that they preferred to have vaginal delivery (VD), while 148 (18.6%) said that they preferred to have CD. The mean age of pregnant women that they preferred CD was 24.86±4.77 years and the mean age of pregnant women that they preferred VD was 23.75±4.35 years. 

There was a significant difference between them in terms of age (p=0.006). As we observe the pregnant women in first pregnancy prefer VD 4.47-fold more than CD. In this study after used of univariate logistic regression model we observed statistically significant relation between women’s preference for CD and the following variables: educational level (p<0.001), gestational age (p<0.001); age (p=0.005), educational level in spouse of pregnant women (p=0.008); local residence (p=0.025), physician’s advice (p<0.001), fear of delivery (p<0.001) and safety of the baby (p=0.005). 

But the relation between women’s preference and the following variables was not statistically significant according to univariate logistic regression model: age (p=0.093), occupation, in pregnant women (p=0.916), occupation in Spouse of pregnant women (p=0.05) and planned pregnancy (p=0.336) (Table I). 

At the end we evaluated the relation between different variables and women’s preference using multivariate logistic regression model with forward method. Variables with significant relations were as follows: educational level, gestational age in pregnant women; age in spouse of pregnant women, physician’s advice and fear of delivery (Table II). There was a significant relation between women’s preference for CD with different educational levels of their (p<0.001) and different durations of spouse′s age of pregnant women (p<0.001) but there wasn’t significant relation between women’s preference for CD and different duration of gestational age (p<0.079) (Table III).

**Table I:** Odds ratio (OR) estimates of women’s preference for CD based on the univariate logistic regression model

**Variables**		**Type of preference delivery**			**OR (95% CI)**
		**Cesarean (N=148)**	**Vaginal (N=649)**	**Total (N=797)**	
**Pregnant women variables**					
Age					(0.92, 2.87)
	≤ 30y	130	598	728	Reference
	> 30y	18	51	69	1.62
Educational level					(1.43, 3.02)
	< Diploma	49	329	378	Reference
	≥ Diploma	99	320	419	2.08
Occupation					(0.49, 2.2)
	Housewife	139	611	750	Reference
	Employee	9	38	47	1.04
Fear of VD/CD					(9.68, 22.82)
	No	63	595	658	Reference
	Yes	85	54	139	14.87
Gestational age					(1.66, 4.17)
	< 37w	114	583	697	Reference
	≥ 37w	34	66	100	2.64
**Spouse of pregnant women variables**					
Age					(1.19, 2.76)
	≤ 30 y	109	542	651	Reference
	> 30 y	39	107	146	1.81
Educational level					(1.13, 2.32)
	< Diploma	69	380	449	Reference
	≥ Diploma	79	269	348	1.62
Occupation					(0.997, 2.6)
	Self-employed	121	570	691	Reference
	Employed	27	79	106	1.61
**Other variables**					
Local residence					(1.06, 2.27)
	Rural	45	262	307	Reference
	urban	103	387	490	1.1
Safety of the baby*					(0.19, 0.76)
	No	133	550	683	Reference
	Yes	9	99	108	0.38
Physician’s advice					(2.13, 6.56)
	No	124	617	741	Reference
	Yes	24	32	56	3.73
Planned pregnancy*					(0.27, 1.57)
	Planned	141	597	738	Reference
	Unplanned	6	39	45	0.65

* Some data were missing in this variable

**Table II:** Odds ratio (OR) estimates of women’s preference for CD based on the multivariate logistic regression model

**Variables**	**β**	**OR**	**95%CI**	**p-value**
Pregnant women educational level	1.06	2.89	(1.78, 4.69)	<0.001
Gestational age	0.878	2.41	(1.34, 4.33)	0.003
Spouse′s age	0.889	2.43	(1.42, 4.17)	0.001
Fear of VD/CD	3.17	23.78	(14.5, 39.13)	<0.001
Physician’s advice	2.05	7.76	(3.9, 15.5)	0.001

**Table III:** Odds ratio (OR) of developing women’s preference for CD according to the educational level, gestational age of pregnant women and their Spouse′s age

**Variables**				**Type of preference delivery**			**OR (95% CI)**
				**Cesarean (N= 148)**	**Vaginal (N=649)**	**Total (N=797)**	
Pregnant women educational level							
	Illiterate			5	12	17	Reference
	Elementary			28	152	180	0.44
							(0.14 , 1.35)
	Junior high school			16	165	181	0.23
							(0.07 , 0.74)
	Senior high school			69	241	310	0.69
							(0.23 , 2.02)
	College			30	79	109	0.91
							(0.3 , 2.81)
Gestational age							
		<16 w		20	135	155	Reference
		16-24 w		38	184	212	1.39
							(0.78 , 2.5)
		25-32 w		44	179	223	1.66
							(0.94 , 2.95)
		≥33 w		46	151	197	2.06
							(1.16 , 3.65)
Spouse′s age							
			>25 y	16	147	163	Reference
			25-29 y	77	361	438	1.96
							(1.11 , 3.47)
			30-34 y	37	98	135	3.47
							(1.8 , 6.58)
			35-39 y	11	28	39	3.61
							(1.52 , 8.59)
			≥ 40 y	7	15	22	4.29
							(1.52 , 12.07)

## Discussion

According to the results of this study, 81.4% of pregnant women in first pregnancy said that they preferred to have VD by the end of the pregnancy period while 18.6% of them preferred to have CD. In two studies that conducted in Hong Kong and Norway, 16.8% and 2.4% of nulliparous women said they would prefer for their baby to be delivered by Cesarean (31, 33). In Mohammadbeigi *et al* that conducted in south of Iran (Shiraz) 50.7% of nulliparous women preferred CD but in Mohammadpour *et al* study which conducted in northwest of Iran (Maragheh) 29.6% of nulliparous women preferred CD (42, 43). 

The CD preference rate in this study and some mentioned studies (especially studies conducted in Iran) are higher than of 15% that suggested by WHO (9). In this study, after using of Multivariate logistic regression model, we observed a positive relation between the women’s preference for CD and their educational level. In Fuglenes *et al* and Faisal *et al* studies, there was a positive relation between pregnant women’s preference for CD and their educational level (33, 34). In Hsu *et al* and Karlstrom *et al* studies, women with lower educational levels had a higher preference for Cesarean (a negative relation) (11, 29). Some studies did not report any significant relation between women’s preference for CD and their educational level (35, 44-46). 

According to the result of this study and some mentioned studies it seems that the educational level of women can probably be one of the factors that may affect the women′s preference for CD, however this relation didn’t observe in some studies. We observed a significant relationship between women′s preference for CD and gestational age. In Pang *et al* study, no significant relation between women′s preferences for CD and gestational age was reported (31). As table III shows, odds ratio of preference for CD increased with increase of gestational age but these differences were not significant. One study was conducted among nulliparous Hong Kong Chinese women showed that significantly more women who preferred CS at 20 week of gestation changed to VD at 37 weeks of gestation than vice versa (36). According to this conflict it seems more investigations are needed to do about relation between gestational age and preference for CD. 

In this study, we observed a significant relationship between women′s preference for CD and age of their spouse. In Chu *et al* study observed that women who had older spouse want more likely to have CD (35). Although in this study and Chu *et al* study, a significant relationship was observed between women′s preference for CD and age of their spouse, but it seems more investigation is needed about this relationship (35). In this study, we observed a significant relationship between women′s preference for CD and physician’s advice. The results of pang *et al* study show that 5.8% of pregnant women prefer Cesarean because of Physician’s advice CD (36).

With attention to pregnant women′s condition, physicians may advice CD to some pregnant women, for example when the baby is in a breech position and can’t turn, when placenta has problems and so on. Fear of delivery is another factor that had relation to women′s preference for CD. A significant relation between fear of delivery and women′s preference for CD was observed in Nieminen *et al* study (26).

Fear of delivery in some studies reported as an effective factor in women′s preference for CD (33, 47). Results of this study on women′s preference for CD are similar to findings by others regarding fear of childbirth, perceived risks of VD, a wish to avoid maternal trauma and optimizing fetal well-being (24, 25, 27, 48-51). We suggest that further studies be undertaken to examine factors influencing women’s childbirth preferences in more detail and prospectively (especially women in first pregnancy). One of the major advantages of present study was that we used of logistic regression model to control effect of confounding variables in presence of other variables but one of the limitations of this study must be highlighted. This was a cross-sectional study which limits considerations regarding causality, because in cross-sectional study the choice was only assessed at a point of time.

## Conclusion

Most of women in this study preferred to have a VD but preference rate for CD exceeded 15% that was suggested by WHO. Various factors influenced women to prefer CD, but in this study; educational level, gestational age in pregnant women; spouse′s age of pregnant women, physician’s advice and fear of delivery were important factors. According to the rate of CD preference in this study suggests the need to counsel women who must choose between VD and CD in first pregnancy.
